# The Association between 25-Hydroxyvitamin D and Hemoglobin A1c Levels in Patients with Type 2 Diabetes and Stage 1–5 Chronic Kidney Disease

**DOI:** 10.1155/2014/142468

**Published:** 2014-08-27

**Authors:** Farshad Kajbaf, Romuald Mentaverri, Momar Diouf, Albert Fournier, Said Kamel, Jean-Daniel Lalau

**Affiliations:** ^1^Department of Endocrinology and Nutrition, University Hospital of Amiens, 80054 Amiens, France; ^2^INSERM Unit 1088, Jules Verne University of Picardie, 80037 Amiens, France; ^3^Bone Biology and Endocrine Division, University Hospital of Amiens, 80054 Amiens, France; ^4^Clinical Research Center, University Hospital of Amiens, 80054 Amiens, France; ^5^Department of Clinical Nephrology, University Hospital of Amiens, 80054 Amiens, France

## Abstract

*Aim*. To examine the relationship between plasma 25-hydroxyvitamin D (25(OH)D) levels and blood hemoglobin A1c (HbA1c) levels in diabetic patients at various stages of chronic kidney disease (CKD).* Methods*. We screened for data collected between 2003 and 2012. The correlation between 25(OH)D and HbA1c levels was studied in patients categorized according to the severity of CKD and their vitamin D status. A multivariate linear regression model was used to determine whether 25(OH)D and HbA1c levels were independently associated after adjustment for a number of covariates (including erythrocyte metformin levels). *Results*. We identified 542 reports from 245 patients. The mean HbA1c value was 6.7 ± 1.0% in vitamin D sufficiency, 7.3 ± 1.5% in insufficiency, and 8.4 ± 2.0% in deficiency (*P* < 0.0001). There was a negative correlation between 25(OH)D and HbA1c levels for the population as a whole (*r* = −0.387, *P* < 0.0001) and in the CKD severity subgroups (*r* = −0.384, *P* < 0.0001 and *r* = −0.333, *P* < 0.0001 for CKD stages 1–3 and 4-5, resp.). In the multivariate analysis, the 25(OH)D level was the only factor associated with HbA1c (*P* < 0.0001). *Conclusion*. 25(OH)D levels were negatively correlated with HbA1c levels independently of study covariates.

## 1. Introduction

In addition to the pivotal role of vitamin D in calcium/phosphorus homeostasis and bone physiology [1, 2], several lines of evidence suggest that vitamin D status may also have a significant role in glucose homeostasis in general [3] and on pathophysiology and progression of metabolic syndrome and Type 2 diabetes in particular [4]. Studies in animals and humans suggest that vitamin D affects insulin secretion and tyrosine phosphorylation of the insulin receptor [3]. Low levels of serum 25-hydroxyvitamin D (25(OH)D) were associated with surrogate measures of insulin resistance, major adverse cardiovascular events, cancers, and all-cause mortality, at least in subjects with metabolic syndrome [5]. Conversely, elevated 25(OH)D levels were associated with a lower risk of incident diabetes [6, 7]. Moreover, it has been reported that most patients with Type 2 diabetes have low 25(OH)D levels and that hemoglobin A1c (HbA1c) levels are negatively correlated with vitamin D status [8].

In view of the above findings, one can hypothesize that vitamin D supplementation decreases insulin resistance and reduces HbA1c levels in patients with diabetes. However, supplementation studies have not unambiguously found that vitamin D favors an improvement in glucose homeostasis parameters [9]. As it is well known that ageing and chronic kidney disease (CKD) are associated with changes in vitamin D metabolism and insulin resistance [10, 11], these studies would have been enriched by taking into account the impact of a number of factors on glucose metabolism, such as age, renal status, and antidiabetic medications but to name a few.

In this regard, antidiabetic medications obviously impact insulin resistance (either directly or indirectly via the reduction in blood glucose levels). In this context, it seems essential to consider the role of metformin (the most frequently prescribed drug to patients with Type 2 diabetes mellitus [12]) in CKD for several reasons: (i) metformin is only contraindicated in severe CKD because it clears four to five times more quickly than creatinine [13], (ii) metformin's impressive cardiovascular protective effects should be of particular value in patients with CKD (who therefore have a high cardiovascular risk) [14], (iii) metformin therapy can potentially be continued in severe CKD as long as the latter one is stable and the dose of metformin is adjusted as a function of the severity of kidney disease [15], (iv) assaying for blood metformin should minimize the risk of metformin accumulation, (v) metformin accumulation is not dangerous per se [16], and (vi) metformin may even be protective in lactic acidosis caused by concomitant conditions [17].

In our institution, we continue metformin administration beyond the limit stated in the current guidelines by applying a pragmatic metformin dose reduction in our patients with low estimated glomerular filtration rate (eGFR) [18]. This provided us with an opportunity to study the relationship between 25(OH)D and HbA1c levels after adjusting for some covariates (including eGFR and erythrocyte metformin levels, the latter one better reflecting potential metformin accumulation than plasma metformin concentrations [19]).

## 2. Methods

### 2.1. Selection of Study Subjects

We systematically reviewed the medical records of all patients with Type 2 diabetes on metformin consulting in our university medical center between 2003 and 2012 (erythrocyte metformin assays were introduced in 2003). In general, the metformin assays had been requested in order to adjust the dose to the patient's renal status or to screen for metformin accumulation [18].

### 2.2. Collection of Biochemical Data

We selected patients for whom data on serum creatinine (to calculate estimated glomerular filtration rate), serum HbA1c, plasma 25(OH)D, and erythrocyte metformin levels were available.

Due to the half-life of vitamin D (of 2-3 weeks, [2]), we only considered HbA1c and metformin samples collected no more than two weeks before or two weeks after the 25(OH)D sample. If several HbA1c and metformin samples had been collected during this four-week interval, we selected those closest in time to the 25(OH)D sample. Likewise, and in view of the close relationship between blood metformin levels and renal status [18], we selected serum creatinine samples collected as close as possible to the metformin sample (and no more than one week before or one week after).

### 2.3. Estimation of the GFR

The eGFR was estimated according to the Modification of Diet in Renal Disease equation, which includes four variables: eGFR (mL/min per 1.73 m^2^) = 175 × (serum creatinine)^−1.154^ × (Age)^−0.203^ × (0.742 if female) × (1.212 if African-American) (conventional units) [20]. The patients were then classified into five CKD stages: >90 mL/min per 1.73 m^2^ (CKD stage 1), from 90 to 60 mL/min per 1.73 m^2^ (CKD stage 2), from 60 to 30 mL/min per 1.73 m^2^ (CKD stage 3), from 30 to 15 mL/min per 1.73 m^2^ (CKD stage 4), and <15 mL/min per 1.73 m^2^ (CKD stage 5).

### 2.4. Data Analyses

After classification by CKD stage, the patients were also divided as a function of their vitamin D status: sufficiency (plasma 25(OH)D ≥30 ng/mL), insufficiency (21–29 ng/mL), and deficiency (i.e., ≤20 ng/mL) [21]. A multivariate analysis was performed to establish whether or not there was an independent relationship between vitamin D status and HbA1c levels after adjusting a priori for age, gender, renal status, and erythrocyte metformin levels.

### 2.5. Analytical Methods

Creatinine levels were measured using a colorimetric assay. 25(OH)D levels were determined using chemiluminometric immunoassays (Liaison 25(OH) Vitamin D Total CLIA, which measures both D_2_ and D_3_; Diasorin, Stillwater, MN; CV: 6.1%). An ion-exchange chromatography method (Variant II Turbo, Bio-Rad, Hercules, CA) was used to assay HbA1c, according to the manufacturer's instructions. Metformin levels were measured in duplicate in the same laboratory using reverse-phase high-performance liquid chromatography with diode-array detection, according to the method described by Lacroix et al. [22].

The erythrocyte level measurement technique has been described in detail elsewhere [19]. The results are expressed as the concentration of basic metformin. The assay's limit of detection was 0.03 mg/L.

### 2.6. Statistical Analysis

For the groups of patients at each CKD stage, the mean ± standard deviation (SD) of 25(OH)D, HbA1c, erythrocyte metformin levels, and age was calculated. The demographic characteristics of the various CKD stages and vitamin D status groups were compared in an analysis of variance (ANOVA). The correlation between 25(OH)D and HbA1c levels was studied in the whole study population and in two separate subgroups of patients with nonsevere CKD (stages 1–3) or severe CKD (stages 4-5) by determining the coefficient of correlation *r*. A multivariate linear regression model with backward selection was used to assess the independence of the association between 25(OH)D and HbA1c levels after adjustment for other study variables.

The threshold for statistical significance was set to *P* ≤ 0.05. All statistical analyses were performed with SAS software (version 9.2, SAS Institute Inc., Cary, NC).

## 3. Results

### 3.1. Demographic and Clinical Characteristics

We identified a total of 542 reports in 245 patients meeting the selection criteria (mean ± SD age: 65 ± 11; male/female gender 342/200, 63%/37%), of whom 80 had 2 or more blood samples during a regular monitoring with a mean interval of 10 months. Patients enrolled in this study did not receive 1,25-dihydroxyvitamin D3 (or one of its active analogs). The population's main demographic and clinical characteristics (including the proportions of patients at CKD stages 1 to 5) are summarized in Table 1.

About 50% of the patients were at CKD stage 3. Stage 5 patients differed significantly from stage 1–4 patients: they were younger, with a lowest mean HbA1c value, a much higher mean 25(OH)D level (around twice that of stage 1 patients), and a much higher mean erythrocyte metformin level (two or threefold greater than in the other subgroups) (*P* < 0.0001). Only 3 patients were on dialysis in this group.

### 3.2. Vitamin D and HbA1c Status

The vitamin D status study groups differed significantly in terms of the eGFR, HbA1c, and 25(OH)D values. These results are in accordance with those obtained from the CKD groups. The group with vitamin D sufficiency had the lowest mean HbA1c value.

The mean HbA1c values were 8.4 ± 2.0% in the subgroup with vitamin deficiency, 7.3 ± 1.5% in the “insufficiency” group, and 6.7 ± 1.0% in the “sufficiency” group (*P* < 0.0001).

### 3.3. Correlation Studies and Multivariate Analysis

**Figure 1:**
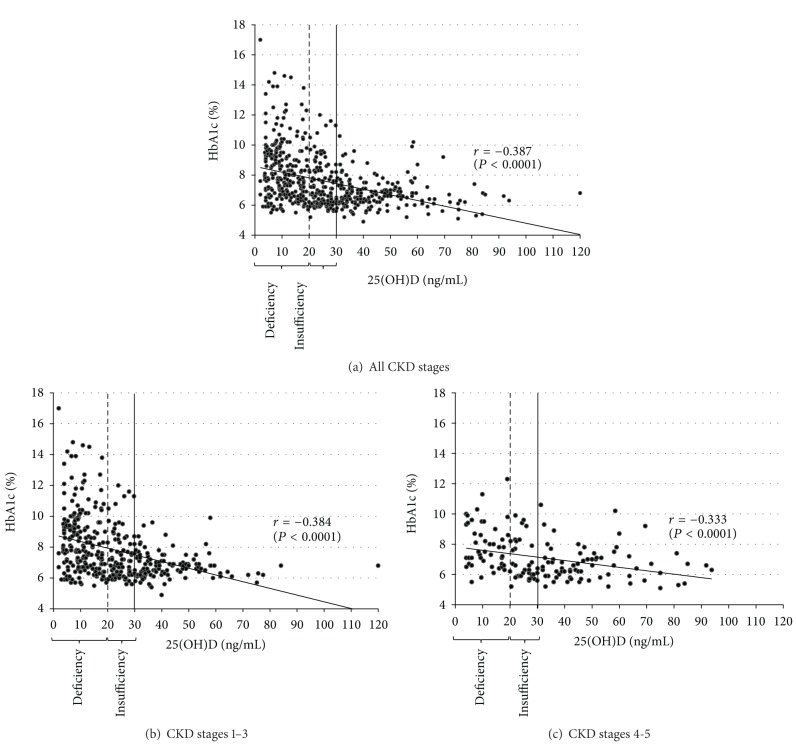
The correlation between 25-hydroxyvitamin D and HbA1c levels in patients with Type 2 diabetes at various stages of chronic kidney disease (correlation coefficient *r*).

There was a negative correlation between 25(OH)D and HbA1c levels for the study population as a whole (*r* = −0.387, *P* < 0.0001) and in the CKD severity subgroups (*r* = −0.384, *P* < 0.0001 and *r* = −0.333, *P* < 0.0001 for CKD stages 1–3 and 4-5, resp.) (Figure 1).

In a multivariate analysis, the 25(OH)D level was the only factor independently associated with HbA1c after controlling for other study variables, (*P* < 0.0001).

## 4. Discussion

In accordance with previous studies [23–26], our present results revealed a significant, negative correlation between 25(OH)D levels and HbA1c values. However, the presence of a correlation does not necessarily mean that vitamin D has a positive impact on glucose homeostasis. Indeed, various factors may influence the relationship between vitamin D and HbA1c. One of the strengths of the present study is inclusion of covariates in the analysis of the association between 25(OH)D and HbA1c. It is well known that some of these covariates (age, gender, and renal status) are influencing vitamin D status [27]. Here, we further refined the analysis of the association between vitamin D and HbA1c by taking account of erythrocyte metformin levels, since (i) the latter one may rise as renal function declines and (ii) this drug is known to influence HbA1c levels [28].

Although the ANOVA revealed a significant difference in the age between the CKD stage subgroups (*P* < 0.0001), most of the study population was middle-aged or elderly (under 70). Most of the patients had moderate to severe CKD (with around 75% at stages 3 to 5). On average, CKD stage 5 patients were younger than other CKD groups (e.g., almost ten years younger than the CKD stage 4 group), suggesting that the onset of CKD had occurred earlier in life in the CKD stage 5 patients.

It is noteworthy that the mean HbA1c value was lower in CKD stage 5 patients than in CKD stage 1–4 patients (6.3% and 7.5%, resp.). This difference must be considered in the light of 25(OH)D levels: the mean 25(OH)D values in the population as a whole and in the CKD stage 1–4 patients were around 25 ng/mL (corresponding to insufficiency), whereas the value in CKD stage 5 patients was almost twice as high (40.6 ng/mL, corresponding to sufficiency). In other words, the CKD stage 5 patients had a particular profile, with an HbA1c value below 6.5% and the absence of vitamin D insufficiency. The results are satisfying from a clinical viewpoint and may reflect our policy of monitoring vitamin D status in patients with severe CKD (one of us (AF) has systematically used 25-OH vitamin D (cholecalciferol, 100 000 U every trimester) for early preventive treatment of secondary hyperparathyroidism in CKD stages 3–5). These observations may suggest a beneficial effect of vitamin D on HbA1c levels. The significantly greater erythrocyte metformin levels in CKD stage 5 patients were taken into account by our multivariate analysis. Consequently, favorable vitamin D status cannot be related to high blood erythrocyte metformin levels. Furthermore, we did not observe any correlation between HbA1c and metformin levels.

However, there are several possible difficulties when seeking to accurately assess long-term glycaemic status according to the HbA1c level in severe CKD: (i) severe CKD is characterized by peripheral insulin resistance [29]; (ii) HbA1c can be carbamylated by isocyanic acid (a reactive form of cyanate formed by the spontaneous dissociation of urea) [30]; and (iii) lifespan of erythrocytes is shortened by up to around one-third in haemodialysis patients [31], due to a combination of factors (vitamin deficiencies, iron deficiency, inflammation [32], the toxic uremic milieu [31], defective synthesis and secretion of erythropoietin [33], and hemolysis [31]). Taken as a whole, these factors may lead to underestimation of the HbA1c level.

Hence, our study results show that despite the presence of insulin resistance, HbA1c levels were the lowest in severe CKD. The carbamylation process did not impact the glycation of HbA1c, as measured by our method [30]. The shortened lifespan of erythrocytes may be a critical factor. Indeed, the HbA1c level was lower in severe CKD than in earlier stages. Nevertheless, it is important to note that the correlation coefficients in the severe CKD and the nonsevere CKD subgroups were almost exactly the same.

The present study had four limitations: (i) the effect of vitamin D on glucose metabolism was not studied directly, (ii) the study was retrospective, (iii) the relatively small number of patients in CKD stages 1 and 5, and (iv) the possible impact of antidiabetic agents other than metformin was not taken into account (although these agents are harder to be assayed in blood than metformin).

## 5. Conclusion

In diabetic patients, at various CKD stages, 25(OH)D levels were negatively correlated with HbA1c values. This association persisted after controlling for covariates such as age, gender, and erythrocyte metformin levels. These covariates must be taken into account when studying the effect of vitamin D supplementation on glucose metabolism in randomized trials.

## Figures and Tables

**Table 1:** Characteristics of the study patients (mean ± standard deviation, *P* value in an ANOVA).

Status	Number of reports, (%)	Age, years	eGFR, mL/min per 1.73 m^2^	HbA1c, (%)	25(OH)D, ng/mL	25(OH)D >30 ng/mL, %	Erythrocyte metformin, mg/L
All reports	542	65.0 ± 11.2	44.3 ± 23.8	7.6 ± 1.8	25.7 ± 18.3	34.7	1.43 ± 1.45
By CKD stage:							
Stage 1	27 (5.0)	59.9 ± 12.6	103.9 ± 11.1	7.5 ± 1.6	19.7 ± 15.9	22.2	0.91 ± 0.60
Stage 2	95 (17.5)	63.4 ± 12.0	72.0 ± 8.7	7.9 ± 1.9	22.7 ± 16.3	28.6	1.06 ± 0.61
Stage 3	268 (49.4)	66.8 ± 10.1	42.9 ± 8.3	7.8 ± 1.9	23.6 ± 16.8	30.6	1.31 ± 0.92
Stage 4	105 (19.4)	66.3 ± 2.0	22.3 ± 4.3	7.4 ± 1.4	28.7 ± 19.2	39.0	1.68 ± 1.99
Stage 5	47 (8.7)	57.8 ± 10.9	9.3 ± 3.1	6.3 ± 1.2	40.6 ± 21.6	68.1	2.58 ± 2.78
*P* value	—	*P* < 0.0001	*P* < 0.0001	*P* < 0.0001	*P* < 0.0001	*P* < 0.0001	*P* < 0.0001
By vitamin D status:							
Sufficiency	188 (34.7)	65.6 ± 10.9	38.3 ± 23.1	6.7 ± 1.0	45.9 ± 14.9	—	1.39 ± 0.97
Insufficiency	109 (20.1)	63.4 ± 11.6	42.5 ± 22.4	7.3 ± 1.5	24.8 ± 2.7	—	1.31 ± 0.87
Deficiency	245 (45.2)	65.3 ± 11.3	49.6 ± 23.8	8.4 ± 2.0	10.7 ± 4.8	—	1.51 ± 1.89
*P* value	—	NS	*P* < 0.0001	*P* < 0.0001	*P* < 0.0001	—	NS

## Abbreviations

25(OH)D: 25-hydroxyvitamin D
HbA1c: Hemoglobin
CKD: Chronic kidney disease
eGFR: Estimated glomerular filtration rate.
